# Management of early-stage HER2-positive breast cancer and attitudes towards HER2DX test in Spain: insights from a nationwide survey

**DOI:** 10.1007/s12094-024-03409-4

**Published:** 2024-04-23

**Authors:** Olga Martínez-Sáez, Javier Cortés, Eva Ciruelos, Mercedes Marín-Aguilera, Gloria González, Laia Paré, Adriana Herrera, Patricia Villagrasa-González, Aleix Prat, Miguel Martín

**Affiliations:** 1 Translational Genomics and Targeted Therapies in Solid Tumors, August Pi i Sunyer Biomedical Research Institute (IDIBAPS), Barcelona, Spain; 2 Department of Medical Oncology, Hospital Clinic of Barcelona, Barcelona, Spain; 3 Department of Medicine, University of Barcelona, Barcelona, Spain; 4 International Breast Cancer Center, Pangaea Oncology Quironsalud Group, Barcelona, Spain; 5 Faculty of Biomedical and Health Sciences, Department of Medicine, Universidad Europea de Madrid, Madrid, Spain; 6 Department of Medical Oncology, Hospital Universitario, 12 de Octubre, Madrid, Spain; 7 HM Hospitales, Madrid, Spain; 8 SOLTI Group, Barcelona, Spain; 9 Reveal Genomics, Barcelona, Spain; 10 Adelphi Targis, Barcelona, Spain; 11 Institute of Oncology (IOB)-Hospital Quironsalud, Barcelona, Spain; 12 Department of Medical Oncology, Hospital Gregorio Marañón, Madrid, Spain; 13 CIBERONC, Centro de Investigación Biomédica en Red de Cáncer, Madrid, Spain; 14 GEICAM, Grupo Español de Investigación en Cáncer de Mama, Madrid, Spain

**Keywords:** HER2, Breast cancer, Neoadjuvant, Trastuzumab, Pertuzumab, T-DM1, Genomic, HER2DX

## Abstract

**Purpose:**

This study aimed to investigate the current therapeutic management of patients with early-stage HER2-positive (HER2+) breast cancer in Spain, while also exploring the perceptions surrounding HER2DX in terms of its credibility, clinical relevance, and impact on therapeutic decision-making. Understanding these aspects is crucial for optimizing treatment strategies and enhancing patient outcomes in the context of HER2+ breast cancer.

**Methods:**

An online questionnaire was conducted by an independent third-party between April and May 2022 across 70 medical oncologists highly specialized in breast cancer management in Spain. The survey included 37 questions regarding treatment decision making in HER2+ early breast cancer.

**Results:**

The management of patients with HER2+ early breast cancer exhibited a high degree of heterogeneity. Among the interviewed oncologists, 53% would recommend upfront surgery for node negative tumors measuring 1 cm or less. Interestingly, 69% and 56% of interviewers were open to deescalate the duration of adjuvant trastuzumab in pT1a and pT1b N0 tumors, respectively. Certain clinicopathological characteristics, such as high grade, high Ki-67, and young age, influenced the decision to prescribe neoadjuvant treatment for patients with clinical stage 1 disease. In cases where neoadjuvant treatment was prescribed for cT1-2 N0 tumors, there was a wide variation in the choice of chemotherapeutic and anti-HER2 regimens. Regarding the use of adjuvant trastuzumab emtansine (T-DM1) in patients with residual disease after neoadjuvant therapy, there was diversity in practice, and a common concern emerged that T-DM1 might be overtreating some patients. HER2DX, as a diagnostic tool, was deemed trustworthy, and the reported scores were considered clinically useful. However, 86% of interviewees believed that a prospective trial was necessary before fully integrating the test into routine clinical practice.

**Conclusion:**

In the context of early-stage HER2+ breast cancer in Spain, a notable diversity in therapeutic approaches was observed. The majority of interviewed medical oncologists acknowledged HER2DX as a clinically valuable test for specific patients, in line with the 2022 SEOM-GEICAM-SOLTI clinical guidelines for early-stage breast cancer. To facilitate the full integration of HER2DX into clinical guidelines, conducting prospective studies to further validate its efficacy and utility was recommended.

**Supplementary Information:**

The online version contains supplementary material available at 10.1007/s12094-024-03409-4.

## Introduction

HER2-positive (HER2+) breast cancer accounts for approximately 20% of all breast cancers and is responsible for a substantial proportion of deaths. In the early-stage (neo)adjuvant chemotherapy plus the anti-HER2 trastuzumab has consistently shown improvements in survival rates [1]. However, there is considerable clinical and biological diversity among patients, impacting their prognosis, and treatment outcomes [2, 3].

Various strategies have been explored to either increase or decrease systemic therapy in early-stage HER2+ breast cancer adapting the therapy to the risk of the patient, aiming to enhance survival and quality of life. These strategies have been heterogeneously implemented in diverse ways [4], such as reducing the intensity and duration of chemotherapy and trastuzumab [5–7], further blocking the HER2 pathway with pertuzumab [8] or neratinib [9], and switching adjuvant trastuzumab to trastuzumab emtansine (T-DM1) for patients who do not achieve a pathologic complete response (pCR) after initial neoadjuvant therapy [10]. Nevertheless, it is evident that many early-stage HER2+ breast cancer patients can be successfully treated with chemotherapy and trastuzumab alone [1], raising concerns about the risk of over-treatment. Thus, the list of decision points in the modern management of early stage HER2+ breast cancer is long, and a “one-size-fits-all” approach to therapy is now outdated.

Several factors beyond tumor burden play a role in determining patients’ prognosis and treatment response for early-stage HER2+ breast cancer. These factors include the hormone receptor status, the intrinsic molecular subtypes of breast cancer [11–13], or the percentage of stromal tumor-infiltrating lymphocytes (TILs) [13–15], all of which have been associated with treatment response and survival. However, current decisions regarding treatment escalation or de-escalation are still primarily based on traditional parameters like tumor size, lymph node involvement, hormone receptor expression, and response to neoadjuvant therapy (pCR achievement or not). Therefore, the development of an objective tool that integrates these various variables can outperform single features and be highly valuable in guiding therapy decisions for early-stage HER2+ breast cancer.

To this end, the HER2DX assay has been developed and validated [16–25]. HER2DX genomic test [17] is a single 27-gene expression and clinical feature-based classifier which provides two independent scores to predict both long-term prognosis and likelihood of pCR in HER2+ early breast cancer treated with trastuzumab based therapy. The assay integrates biological information tracking immune response, luminal differentiation, tumor cell proliferation and expression of the HER2 17q12-21 chromosomal amplicon, including the *ERBB2* gene, with clinical information (i.e., tumor size and nodal status) [17]. Overall, HER2DX is the first combined prognostic score based on clinicopathological and genomic variables in early-stage HER2+ breast cancer, which identifies a substantial proportion of patients who might not need additional therapies, such as pertuzumab, neratinib, or T-DM1 because of their favorable survival outcomes with chemotherapy and trastuzumab. In addition, HER2DX can identify patients with high-risk disease, who might need additional anti-HER2 therapies beyond trastuzumab, helping to guide clinical decisions in the daily practice. The current SEOM-GEICAM-SOLTI clinical guidelines for early-stage breast cancer support its use in selected clinical situations (level of recommendation IIB) [26].

In this national, web-based survey, medical oncologists were asked about how they approach early-stage HER2+ breast cancer and about their knowledge and opinion regarding the HER2DX assay.

## Methods

### Study design

Adelphi Targis (Barcelona, Spain), at the request of Reveal Genomics, developed and undertook an independent online survey of medical oncologists from April to May 2022. Eligible participants were those who routinely treated patients with breast cancer, constituting at least 20% of their total patient caseload, and possessed prior experience with genomic tests in the context of breast cancer. The comprehensive survey comprised 37 questions, which needed to be completely answered, and required approximately 20 min for completion (**Supplemental Data**).

Respondents were carefully selected from a comprehensive database of health professionals and subsequently contacted directly through email. The data collection process encompassed a wide range of information, including participant demographics, practice settings, years of experience in oncology and specialization in breast cancer, as well as their corresponding autonomous communities. The survey’s content delved into various aspects of the attitudes of breast medical oncologists concerning the management of patients with HER2+ early-stage breast cancer. This included inquiries about their preferences for neoadjuvant treatment versus upfront surgery for small tumors, the use of multiagent chemotherapy versus single-agent regimens, the employment of adjuvant T-DM1, and the duration of adjuvant trastuzumab therapy. Furthermore, participants were asked to provide their perspectives on the credibility and clinical utility of HER2DX. Respondents were remunerated for their participation in this activity. For the complete set of survey questions, please refer to **Table S1**. It is important to note that all data collected were treated anonymously to maintain confidentiality.

The study aimed to examine the present therapeutic approaches for patients diagnosed with HER2+ early-stage breast cancer in Spain. It sought to identify the factors influencing treatment decisions, including tumor and/or patient characteristics. Additionally, the study aimed to assess perceptions about the credibility of the existing evidence with HER2DX, the clinical significance of this tool, and its impact on therapeutic decision-making based on different patient profiles.

### Statistical analysis

In this investigation, an exploratory approach was adopted to gain insights into the subject matter. Descriptive statistics were extensively employed to provide a comprehensive overview of the data collected from the survey. The utilization of bar plots facilitated a visual representation of various key findings.

## Results

### Participant demographics

A total of 70 oncologists from 12 out of 17 (71%) Spanish autonomous communities were invited to take part in the survey, and all of them willingly to participate. The median age of the participants was 46 years (ranging from 32 to 63 years), with a majority of women, comprising 55.7% of the respondents. Most participants (98.6%) practiced in public healthcare centers, and their collective experience in breast cancer averaged at 16.1 years (ranging from 3 to 36 years) (see Table 1). On average, the participants attended to 79.5 breast cancer patients per month, among whom 27.1% were HER2+ .

**Table 1:** Participants characteristics

Age (mean), years old	46
Sex, *n* (%)	
Female	39 (55.7%)
Male	31 (44.3%)
Primary place of work, *n* (%)	
Public practice	69 (98.6%)
Private practice	1 (1.4%)
Size of the hospital^a^	
Group 1	0 (0%)
Group 2	8 (11%)
Group 3	23 (33%)
Group 4	14 (20%)
Group 5	25 (36%)
Professional position, *n* (%)	
Head of the service	3 (4%)
Head of section	7 (10%)
Attending physician	60 (86%)
Experience in clinical oncology, years, *n* (%)	
< 5	1 (1%)
5–14	27 (39%)
15–24	30 (43%)
25–34	9 (13%)
≥ 35	3 (4%)
Experience in breast cancer, years, *n* (%)	
< 5	1 (1%)
5–14	31 (44%)
15–24	29 (41%)
25–34	8 (11%)
≥ 35	1 (1%)
Autonomous community, *n* (%)	
Andalusia	15 (21%)
Madrid	10 (14%)
Catalonia	9 (13%)
Galicia	7 (10%)
Vasc Country	7 (10%)
Valencia	5 (7%)
Estremadura	5 (7%)
Castile and Leon	5 (7%)
Castile-La Mancha	3 (4%)
Aragon	2 (3%)
Murcia	1 (1%)
Navarre	1 (1%)

^a^Definitions of each group are included in supplementary data

### Clinical stage 1 (cT1 N0) setting

**Fig 1:**
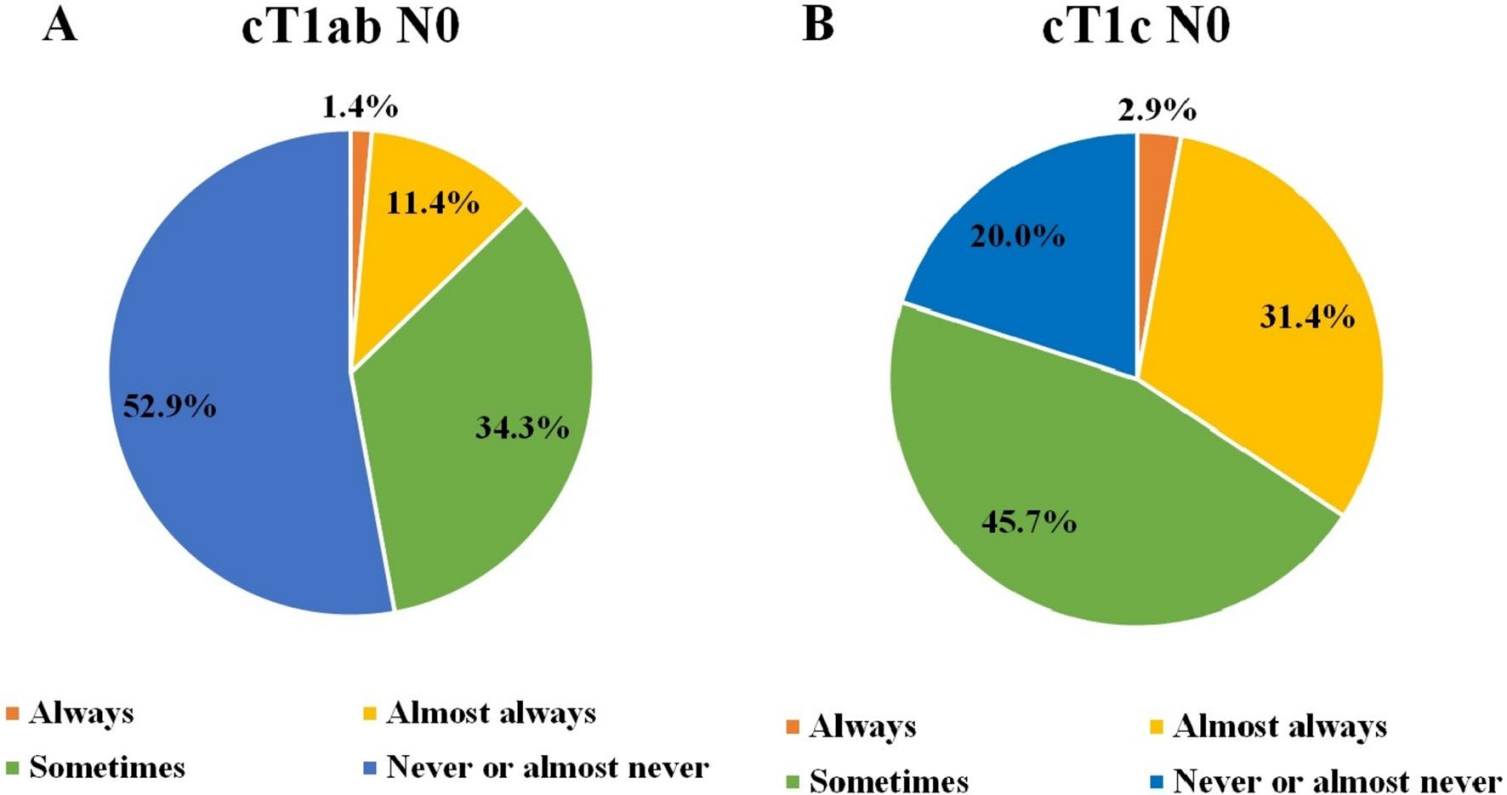
Intention to treat with neoadjuvant therapy based on clinical stage. **A** Clinical stage T1ab N0. **B** Clinical stage T1c N0

**Fig 2:**
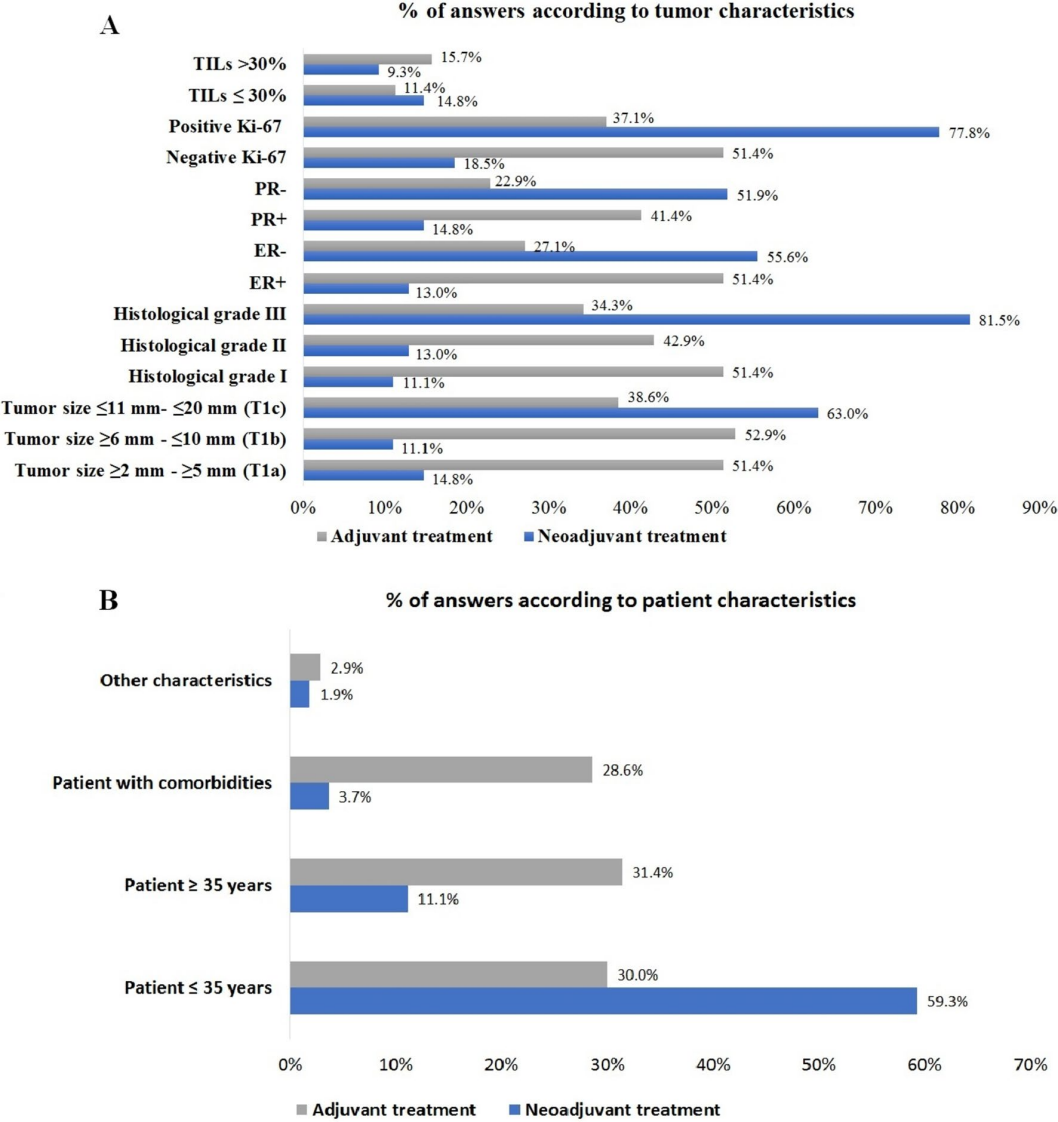
Factors that potentially serve as encouragements for opting for neoadjuvant treatment in stage 1 (cT1 N0) disease. **A** Tumor factors. **B** Patient factors. *RE,* estrogen receptor; *PR,* progesterone receptor; *TILs,* tumor infiltrating lymphocytes. Ki-67 positive was defined as  >  20%; Ki-67 negative was defined as  ≤  20%

Among the interviewed oncologists, 53% stated that they would never or almost never offer neoadjuvant therapy to patients with cT1a/b N0 tumors. Conversely, 34% of the respondents indicated that they would sometimes consider the neoadjuvant approach in such cases, while only 13% stated they would do it always or almost always. For patients with stage cT1c N0 tumors, 20% of the oncologists reported that they would never or almost never use neoadjuvant therapy, 46% would use it occasionally, and 34% expressed that they would always or almost always employ this approach (Fig. 1). Factors that would encourage opting for neoadjuvant treatment in stage cT1 N0 were grade 3, Ki-67 > 20%, tumor size > 1 cm and young age (≤ 35 years old) (Fig. 2).

**Fig 3:**
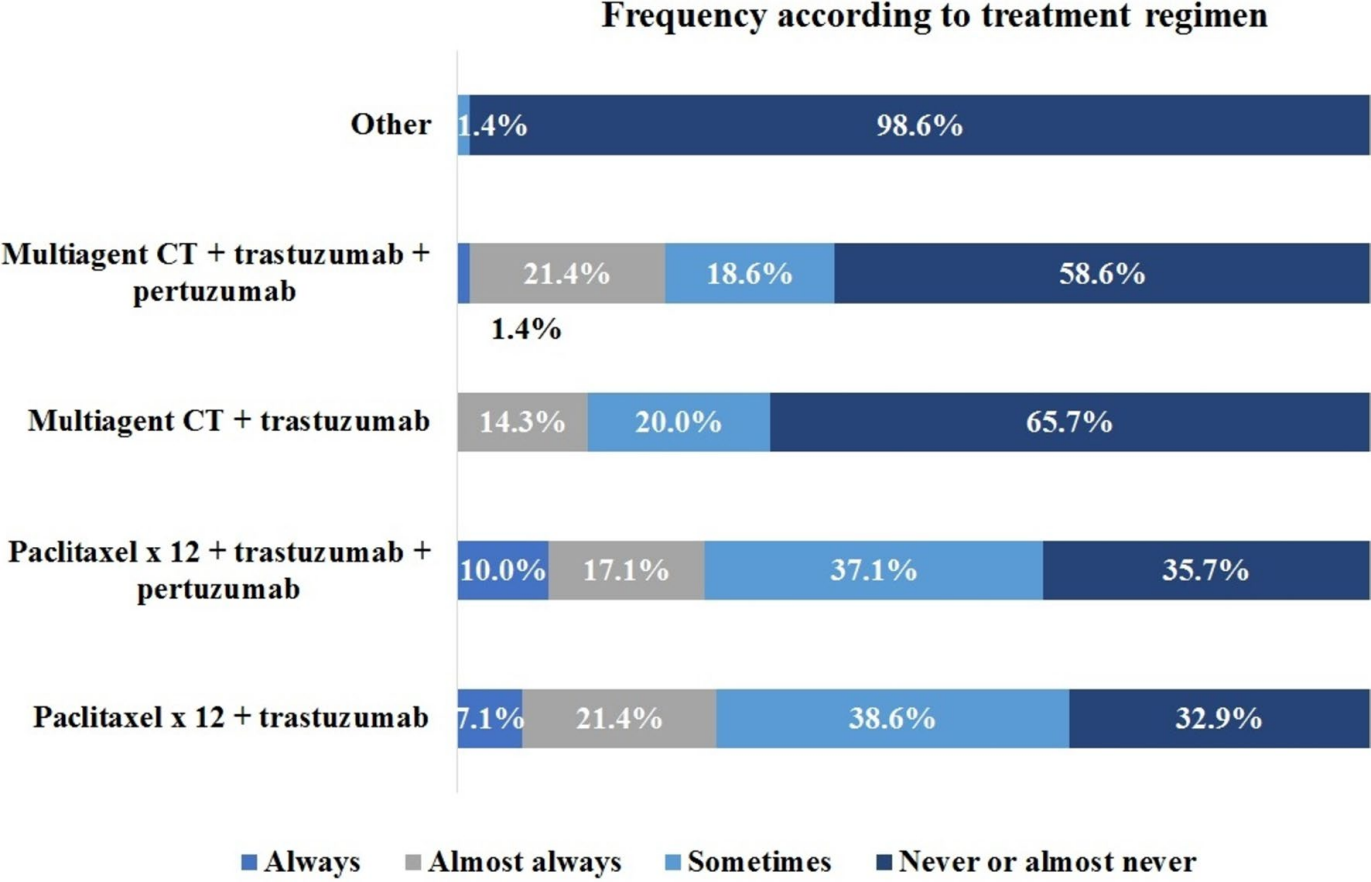
Neoadjuvant therapeutic regimens used in clinical stage T1a/b N0. CT, chemotherapy

In cases of patients with cT1a/b N0 who were treated with a neoadjuvant approach, a significant variability was observed in the prescribed therapeutic regimens (Fig. 3). Regarding multi-agent chemotherapy and dual HER2 blockade, 25.4% of participants would use it always or almost always. Concerning single taxane and dual HER2 blockade, 37.1% of participants would use it always or almost always. Regarding single taxane and trastuzumab, 28.5% of participants would use it always or almost always.

**Fig 4:**
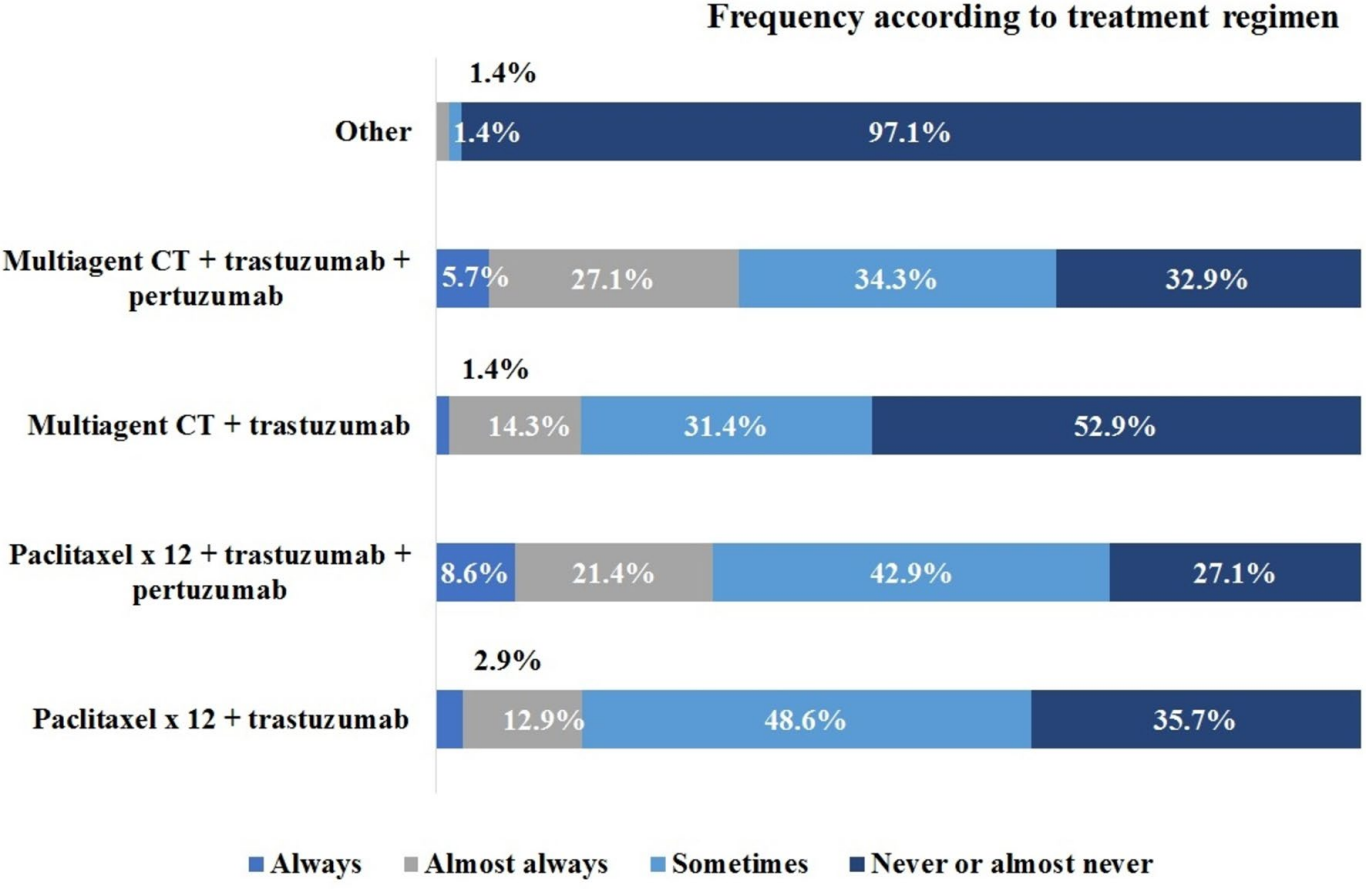
Neoadjuvant therapeutic regimens used in clinical stage T1c N0. CT, chemotherapy.

Among the respondents, even a greater variability in neoadjuvant treatment choices was observed for patients with cT1c N0 compared to those with stage cT1a/b N0 (Fig. 4).

### Pathological stage 1 (pT1 N0)

The mean minimum size to prescribe the APT schema [27] among patients with pT1 N0 who underwent primary surgery was 7 mm (interquartile range 5–10). The respondents indicated that they would consider an adjuvant multi-agent chemotherapy regimen if the tumor displayed certain aggressiveness features. Notably, 87% of the interviewees would contemplate this approach for tumors classified as grade 3, 83% for those with high Ki-67, 67% for tumors larger than 10 mm, 64% for patients aged ≤ 35 years old, and 61% for estrogen receptor-negative tumors (Fig. S1).

### Clinical stage cT2 cN0, or cN1

Among the interviewees, 66% would offer multiagent neoadjuvant chemotherapy with trastuzumab to patients with cT2 N0, while 70% would do so for those with N1. In contrast, 34% would treat patients with single-agent paclitaxel for stage cT2 N0, and 30% for cN1, respectively. The most significant clinicopathological factors that supported the use of multi-agent chemotherapy in these scenarios were histological grade 3, Ki-67 > 20%, and tumor size > 30 mm (**Figs. S2, S3**).

### Duration of adjuvant trastuzumab

**Fig 5:**
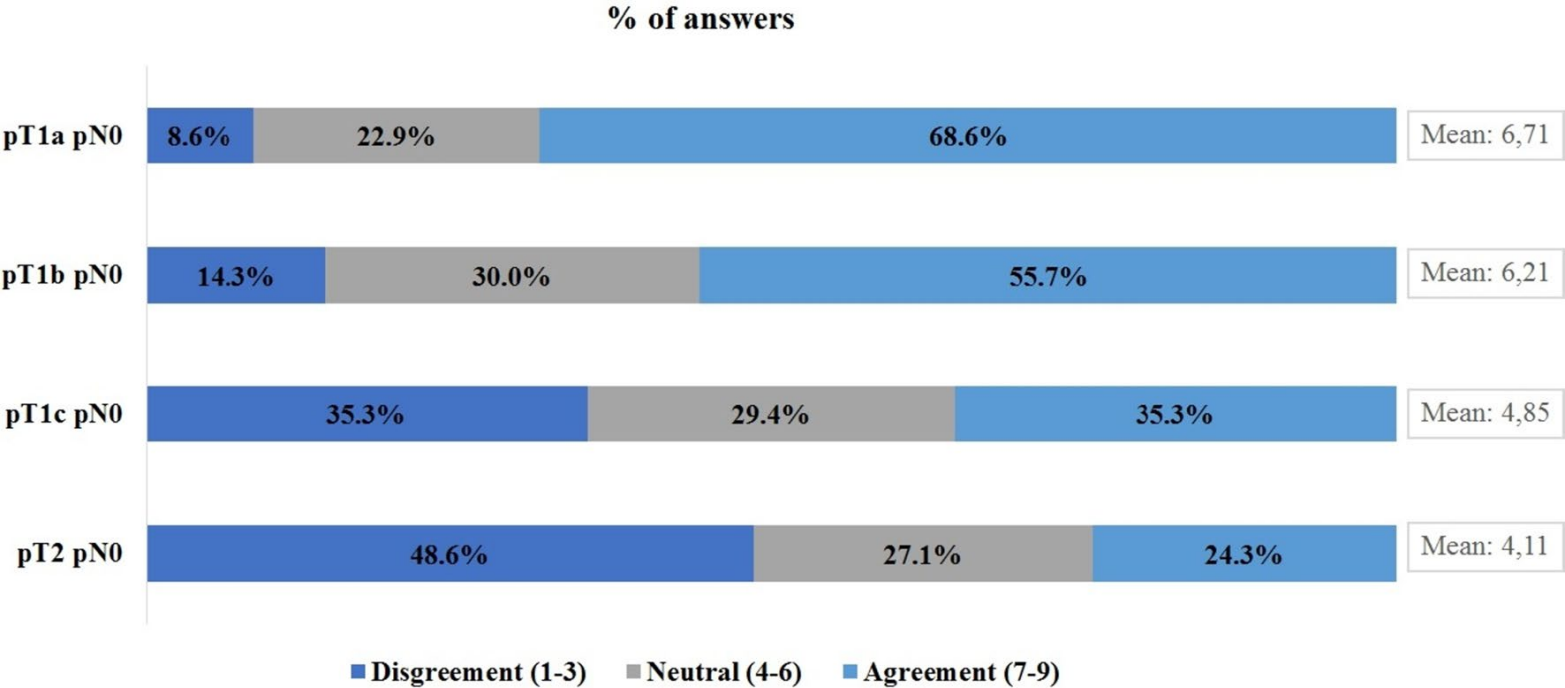
Percentage of agreement regarding the use of 6-month adjuvant trastuzumab according to pathological stage

The interviewed oncologists were asked an agreement question regarding the use of 6 months of trastuzumab in patients with pT1-2 N0 tumors, with a rating scale from 1 to 9 (where 1 indicated complete disagreement, and 9 represented the maximum agreement). The findings revealed that 69% of the interviewees agreed with the use of 6 months of adjuvant trastuzumab in patients with pT1a N0 tumors, 56% in pT1b N0, 35% in pT1c N0, and 24% in pT2 N0 tumors (Fig. 5).

However, it was found that only 20% of the oncologists acknowledged using 6-month trastuzumab in their daily practice. The most frequently reported reasons for not using it more frequently were as follows: 57% stated that the regimen was not included in clinical guidelines, 41% expressed insecurity with the use of the 6-month schema, 21% believed that trastuzumab was a safe treatment with manageable side effects, and 18% considered that the benefits of the 12-month regimen outweighed the risks.

### Adjuvant T-DM1

Among the interviewees, 63% agreed to offer adjuvant T-DM1 to patients with cT1 N0 and residual disease after neoadjuvant therapy. Meanwhile, 20% held a neutral opinion on its use in this scenario, and 17% disagreed. In cases of patients with cT2 N0 and specific circumstances, such as pCR in the breast with micrometastases in the axilla, only 50% of the respondents would offer adjuvant T-DM1, indicating a lack of high agreement. Furthermore, when the residual disease was HER2-negative, only 34% of the oncologists would treat with T-DM1. Interestingly, a majority of the interviewees (3% always, 7% almost always, and 57% sometimes) felt that they tended to overtreat patients when using adjuvant T-DM1.

#### HER2DX

The study revealed that a considerable number of interviewees, 77% and 74%, respectively, expressed a high level of agreement (score 7–9 on a scale from 1 to 9) regarding the reliability of the risk of recurrence and pCR scores reported by HER2DX. Conversely, 21% and 24% held a neutral opinion (score 4–6) on the risk of recurrence and pCR score, while 4% did not consider the test reliable (score 1–3). Furthermore, a significant majority of oncologists, 79%, 76%, and 67%, believed that pCR score, risk of recurrence score, and *ERRB2* levels, respectively, would have an impact on clinical practice.

The survey highlighted the potential role of HER2DX in guiding treatment decisions. Among the participants, 64% considered that the risk of recurrence score would be helpful in deciding whether to administer neoadjuvant therapy or choose upfront surgery for patients with cT1 N0 tumors. Similarly, 64% believed that the pCR score could aid in this decision-making process. In cases of patients with cT2 N0 tumors, 59% and 57% of the interviewees felt that the risk score and pCR score, respectively, would assist in making such treatment choices. Additionally, 53% of the oncologists believed that HER2DX could help decide between multiagent chemotherapy versus single agent taxane with anti-HER2 therapy for patients with cT2 N0 tumors.

For patients with cT1 N1 tumors, 51% of oncologists thought HER2DX could be useful in determining whether to opt for multi-agent chemotherapy versus single agent taxane plus anti-HER2 therapy, while 44% expressed a similar opinion for cases with cT2 N1 tumors. Regarding the duration of adjuvant trastuzumab, 60% of participants felt that HER2DX would aid in deciding whether to shorten its administration for patients with pT1a/b tumors, and 57% for patients with pT1c tumors. Moreover, for adjuvant therapy decisions, 63% considered that HER2DX could guide the use of adjuvant T-DM1 in selected patients, while 56% believed it could help avoid adjuvant pertuzumab in patients who achieved a pCR. Additionally, 51% considered that HER2DX could assist in omitting chemotherapy in small tumors with a low risk of recurrence.

In general, the majority (66%) of interviewees agreed that HER2DX could be beneficial in making clinical decisions for selected patients. However, 86% felt that a prospective validation of HER2DX was necessary before its implementation in clinical practice. Furthermore, 79% agreed that international guidelines should recommend the use of HER2DX before incorporating it into daily practice. When asked about the first indication for the test, 71% agreed it should be used to guide the de-escalation of trastuzumab duration in some patients, 70% agreed it must assist in the de-escalation of multi-agent chemotherapy in selected patients, 66% considered de-escalation with the use of adjuvant T-DM1 in selected patients as the first indication, and 59% believed the first indication should guide the de-escalation in the use of pertuzumab in selected patients.

## Conclusions

This study aimed to shed light on the therapeutic management of HER2+ early-stage breast cancer in Spain, examining the perspectives of oncologists and their attitudes towards HER2DX. A total of 70 oncologists from 12 out of 17 Spanish autonomous communities participated in the survey. The respondents were primarily women (55.7%) practicing in public centers (98.6%), with an average experience of 16.1 years in breast cancer management.

The study revealed a significant heterogeneity in the therapeutic approach for HER2+ early-stage breast cancer among the interviewed oncologists. Even for tumors measuring less than 1 cm without nodal involvement, 34% of oncologists would consider using neoadjuvant therapy occasionally, while about 53% would not or rarely offer it in this scenario. This discrepancy highlights the diversity in treatment preferences and clinical decision-making. Similarly, for cT1c N0 tumors, the variability increased, with 20% of oncologists never or almost never using neoadjuvant therapy, 46% using it sometimes, and 34% always or almost always employing this approach.

The study identified specific clinicopathological factors that influenced the decision to offer neoadjuvant therapy. In cases of cT1 N0 tumors, factors such as histological grade 3, Ki-67 > 20%, tumor size > 30 mm, and young age (≤ 35 years old) encouraged oncologists to opt for neoadjuvant treatment. These findings suggest that tumor characteristics and patient demographics play a crucial role in treatment decisions.

The research also highlighted considerable variability in the prescribed therapeutic regimens for patients undergoing neoadjuvant treatment. Even for small tumors, cT1a/b N0, and despite guidelines recommendations [28], 25.4% of participants preferred multi-agent chemotherapy with dual anti-HER2 blockade, 37.1% chose single-agent paclitaxel with dual HER2 blockade, while only 28.5% would always or almost always prescribe the APT regimen. Higher variability was observed regarding the chosen systemic treatment in cT1c N0. These numbers illustrate the diversity in the real-world therapeutic approaches for HER2+ early-stage breast cancer. Moreover, they point out entrenched behaviors and resistance to de-implementation strategies that have been described in the medical community [29, 30]. While it is well known that de-escalation strategies will reduce costs and toxicities, risk aversion and prior outcome preference could also explain why physicians do not fully adhere to the new guidelines recommendations [30, 31].

Interestingly, although most of the interviewees agreed with using a shortened schema of trastuzumab in pT1ab N0 tumors, only 20% would really use it in their daily practice. In line with this, 67% felt that they tended to overtreat patients when using adjuvant T-DM1. Insecurity and the non-recognition by clinical guidelines were the most common reasons in this survey to not use 6-month trastuzumab.

HER2DX emerged here as a potential tool to guide and support treatment decisions. In general, most oncologists regarded the risk of recurrence and pCR scores reported by HER2DX as reliable information. Moreover, a significant proportion of participants believed that HER2DX would have an impact on clinical practice, influencing the choice of neoadjuvant therapy or upfront surgery for patients with cT1 N0 or cT2 N0 tumors. Additionally, HER2DX was perceived as useful supporting de-escalation strategies, such deciding between multi-agent chemotherapy versus single-agent taxane with anti-HER2 therapy for cT1-2 N0-1 tumors, choosing the duration of adjuvant trastuzumab in stage I, guiding the use of adjuvant T-DM1 in selected patients, and avoiding adjuvant pertuzumab in patients who achieved a pCR.

While oncologists acknowledged the potential of HER2DX in guiding treatment decisions, they emphasized the desire for prospective validation before integrating it into routine clinical practice. The majority agreed that guidelines should recommend the use of HER2DX before its widespread implementation. As the study demonstrated the variability in therapeutic approaches and the potential benefits of HER2DX, prospective validation can further establish its credibility and support its incorporation into clinical guidelines for the management of HER2+ early-stage breast cancer. In that sense, the DEFINITIVE phase III trial, funded by the European Union with Horizon Europe 2023–2024 work program, will investigate the clinical value of HER2DX by randomizing patients with stage II-IIIA HER2+ breast cancer to be based on HER2DX results or by physician´s choice, and will began enrolling patients in June 2024. The goal of the trial is to de-escalate treatment in specific patients´ populations aiming to increase their quality of life, while maintaining the efficacy outcomes. The trial will engage patient advocates throughout all phases of the study, demonstrating a commitment to incorporating patients’ perspectives. This approach addresses a gap identified in standard practice, as evidenced by a recent prospective survey of 622 European patients with HER2+ breast cancer [32].

The findings from the present study provide valuable insights into the current therapeutic landscape in Spain and highlight the importance of personalized treatment approaches and homogenization guided by reliable genomic tests like HER2DX. It is worth mentioning that the Spanish clinical breast cancer guidelines, published jointly by SEOM-GEICAM-SOLTI in May 2023, recommend (level IIB) the clinical implementation of HER2DX for specific patient cases, and, moreover, the St Gallen 2023 Consensus Guidelines recognized HER2DX as a practice-changing finding [26, 28].

Despite the valuable insights gained from this study on the therapeutic management of HER2+ early-stage breast cancer and the attitudes towards HER2DX, there are certain caveats that should be considered. Firstly, the study’s sample size of 70 oncologists may not fully represent the entire population of healthcare providers in Spain, potentially limiting the generalizability of the findings. Moreover, the study was conducted in a specific timeframe, and treatment practices and opinions may have evolved since then, emphasizing the need for continuous monitoring and reassessment. Furthermore, as with any survey-based study, there could be recall or reporting bias from the participants, affecting the accuracy of the gathered information. Finally, the perspectives of oncologists may not fully reflect patient preferences and experiences, which could also influence treatment decisions. Despite these caveats, this study provides valuable insights into the current landscape of HER2+ breast cancer management and sets the foundation for further research and prospective validation to address these limitations and strengthen the evidence base for personalized treatment strategies.

### Supplementary Information

Below is the link to the electronic supplementary material.

Supplementary file1 (PDF 221 KB)

Supplementary file1 (PDF 440 KB)

Supplementary file1 (Xlsx 20 KB)

## Data Availability

This work was supported by Reveal Genomics (no grant number).
