# Supplementary material for: Management of early-stage HER2-positive breast cancer and attitudes towards HER2DX test in Spain: insights from a nationwide survey
Source: Clin Transl Oncol. 2024 Apr 23;26(8):2060–9. doi: 10.1007/s12094-024-03409-4 (PMC11249709; doi:10.1007/s12094-024-03409-4)
Supplement: Supplementary file 1 — Supplementary file1 (PDF 221 KB) [file 12094_2024_3409_MOESM1_ESM.pdf]

1. ¿En qué provincia trabaja actualmente?
2. Por favor, indique si es usted:
3. Por favor, indique su edad:
4. Por favor, indique la especialidad que ejerce en su práctica clínica actual:
5. En la actualidad, ¿en qué ámbito dedica la mayor parte del tiempo (más del 50%) de su actividad profesional?
6. ¿Cuál es su cargo profesional?
7. Atendiendo a la siguiente clasificación de hospitales, en la actualidad, dentro del ámbito donde realiza su labor profesional mayoritaria ¿cómo definiría el hospital en el que dedica más del 50% de su actividad profesional?
8. ¿Cuántos años de experiencia tiene en el ámbito de la oncología médica?
9. ¿Cuántos años de experiencia tiene en el manejo y tratamiento del cáncer de mama?
10. En su práctica habitual, aproximadamente, ¿a cuántos nuevos pacientes oncológicos atiende usted de media cada año? (piense en pacientes no en visitas)
11. De los nuevos pacientes oncológicos que ha señalado que usted vista de media al año, ¿qué porcentaje de ellos presentan cáncer de mama? (piense en pacientes no en visitas)
**Nuevos pacientes oncológicos /año**
% nuevos pacientes con cáncer de mama al año (respecto al total de nuevos pacientes oncológicos)
12. ¿Tiene experiencia en el uso de plataformas genómicas en cáncer de mama?
13. ¿Cuántos años de experiencia tiene en el uso de plataformas genómicas en cáncer de mama?
14. De las siguientes plataformas genómica y/o test genómicos, ¿puede indicarnos la siguiente información?
15.Pensando únicamente en las pacientes con cáncer de mama en estadios precoces, ¿puede indicarnos cuántas nuevas pacientes ve de media al año? (piense en pacientes no en visitas)
**Nuevas pacientes con cáncer de mama en estadios precoces/año**
16.Nos comenta que al año visita @1 nuevas pacientes con cáncer de mama en estadios precoces, ¿podría hacer una distribución en % según los diferentes subtipos?
17. Si nos centramos en el volumen de nuevas pacientes con cáncer de mama HER2+ que ha señalado visitar al año, ¿puede distribuirlo en % según los diferentes estadios?
18. Si nos centramos en el volumen de nuevas pacientes con cáncer de mama HER2+ en estadio precoz que ha señalado visitar al año, ¿puede distribuirlo en % según los diferentes estadios clínicos?

19.B ¿Qué características del tumor y/o paciente motivan esta decisión? Marque todas las respuestas que apliquen.
19. Pensando en el volumen de nuevos pacientes con cáncer de mama HER2+ estadio clínico cT1 cN0 que visita al año, ¿qué % de estas pacientes irían a neoadyuvancia? ¿Qué % de estas irían a adyuvancia (cirugía primaria)?
20. Pensando en el volumen de nuevos pacientes con cáncer de mama HER2+ estadio clínico cT2 cN0 que visita al año, ¿qué % de estas irían a un régimen u otro de neoadyuvancia?
Estadio cT1 cN0: @1 % Pacientes que van a Neoadyuvancia
Estadio cT1 cN0: @1% Pacientes que van a Adyuvancia (Cirugía primaria)
20.B ¿Qué características del tumor y/o paciente motivan esta decisión? Marque todas las respuestas que apliquen.
**Por favor, cumplimente las siguientes preguntas teniendo en cuenta que las respuestas deben basarse en el ámbito asistencial en el que realiza su labor profesional mayoritaria en @1 (más del 50% de su jornada).**
Estadio cT2 cN0: @1% Pacientes que van a Neoadyuvancia: Paclitaxel + trastuzumab + pertuzumab
Estadio cT2 cN0: @1 % Pacientes que van a Neoadyuvancia: Quimioterapia multiagente + trastuzumab + pertuzumab
21. Pensando en el volumen de nuevos pacientes con cáncer de mama HER2+ estadio clínico cN1 que visita al año, ¿qué % de estas irían a un régimen u otro de neoadyuvancia?
21.B ¿Qué características del tumor y/o paciente motivan esta decisión?. Marque todas las respuestas que apliquen.
**Por favor, cumplimente las siguientes preguntas teniendo en cuenta que las respuestas deben basarse en el ámbito asistencial en el que realiza su labor profesional mayoritaria en @1 (más del 50% de su jornada).**
Estadio cN1: @1% Pacientes que van a Neoadyuvancia: Paclitaxel + trastuzumab + pertuzumab
Estadio cN1: @1 % Pacientes que van a Neoadyuvancia: Quimioterapia multiagente + trastuzumab + pertuzumab
22.Ante una paciente HER2+ y estadio clínico cT1a/b cN0, en la actualidad, ¿pauta terapia neoadyuvante?
23. Ante una paciente HER2+ y estadio clínico cT1a/b cN0, en la actualidad, ¿con qué frecuencia pauta los siguientes regímenes de terapia neoadyuvante?
24.Ante una paciente HER2+ y estadio clínico cT1c cN0, en la actualidad, ¿pauta terapia neoadyuvante?
25. Ante una paciente HER2+ y estadio clínico cT1c cN0, en la actualidad, ¿con qué frecuencia pauta los siguientes regímenes de terapia neoadyuvante?

26. Ante una paciente HER2+ y estadio patológico pT1 pN0 y operada de entrada, ¿en qué situaciones se plantearía pautar un régimen de quimioterapia multiagente? Marque todas las respuestas que apliquen.
**Por favor, cumplimente las siguientes preguntas teniendo en cuenta que las respuestas deben basarse en el ámbito asistencial en el que realiza su labor profesional mayoritaria en @1 (más del 50% de su jornada).**
27.Ante una paciente HER2+ y estadio patológico pT1 pN0, operada de entrada, ¿qué mínimo del tumor invasivo (en mm) le hace pautar paclitaxel+trastuzumab 1 año (APT)?
28. En base a esta nueva evidencia, pensando siempre en el impacto en su práctica clínica real, ¿se plantearía esquemas de tratamiento de trastuzumab 6 meses en los siguientes perfiles de pacientes? Siendo 1, no modifica el esquema el esquema terapéutico en estas pacientes y 9, definitivamente modifica el esquema esquema terapéutico en estas pacientes
**Le presentamos a continuación la publicación de ESMO 2021 (Earl et al.) Este metaanálisis concluye la no inferioridad de trastuzumab adyuvante 6 meses vs 12 meses.**
29. En la actualidad, ¿pauta usted trastuzumab 6 meses ante determinados perfiles de pacientes HER2+?
30. En un perfil de paciente HER2+ estadio clínico cT1 cN0 que ha recibido tratamiento neoadyuvante, pensando en la posibilidad de dar T-DM1 adyuvante en caso de enfermedad residual, ¿cómo impacta este hecho en su práctica clínica? Siendo 1, no modifica nada mi práctica clínica y 9, definitivamente modifica mi práctica clínica
31. En las pacientes HER2+ estadio clínico cT2 cN0 que reciben quimioterapia neoadyuvante basada en anti-HER2 que presentan IHC/ISH HER2-negativo en el tumor residual, ¿pauta en adyuvancia T-DM1?
33. Por favor, pensando siempre en su práctica clínica real, ¿cree que estar sobretratando a muchas pacientes con T-DM1 en adyuvancia?
34. Por favor, pensando en la información que le acabamos de mostrar, ¿cuánto de creíble son para usted cada uno de los siguientes ítems?
35. Por favor, pensando en la información que le acabamos de mostrar, ¿qué relevancia clínica tienen para usted cada uno de los siguientes ítems?
36.Por favor, pensando en la información que le acabamos de mostrar, ¿cuál es su grado de acuerdo con las siguientes afirmaciones relacionadas con HER2DX?
37. Por favor, ante la disponibilidad de HER2DX en su práctica clínica real, ¿qué impacto tendría HER2DX en su toma de decisiones de tratamiento para los perfiles de pacientes indicados?

ACEPTA	Acepto	1
ACEPTA	No acepto	2
P1	Albacete	2
P1	Alicante	3
P1	Almería	4

P1	Álava	1
P1	Asturias	33
P1	Ávila	5
P1	Badajoz	6
P1	Balears, Illes	7
P1	Barcelona	8
P1	Bizkaia	48
P1	Burgos	9
P1	Cáceres	10
P1	Cádiz	11
P1	Cantabria	39
P1	Castellón	12
P1	Ceuta	51
P1	Ciudad Real	13
P1	Córdoba	14
P1	Coruña, A	15
P1	Cuenca	16
P1	Gipuzkoa	20
P1	Girona	17
P1	Granada	18
P1	Guadalajara	19
P1	Huelva	21
P1	Huesca	22
P1	Jaén	23
P1	León	24
P1	Lleida	25
P1	Lugo	27
P1	Madrid	28
P1	Málaga	29
P1	Melilla	52
P1	Murcia	30
P1	Navarra	31
P1	Ourense	32
P1	Palencia	34
P1	Palmas, Las	35
P1	Pontevedra	36
P1	Rioja, La	26
P1	Salamanca	37
P1	Santa Cruz de Tenerife	38
P1	Segovia	40
P1	Sevilla	41
P1	Soria	42
P1	Tarragona	43
P1	Teruel	44
P1	Toledo	45
P1	Valencia	46
P1	Valladolid	47
P1	Zamora	49

P1	Zaragoza	50
CCAA	Andalucía	1
CCAA	Aragón	2
CCAA	Principado de Asturias	3
CCAA	Cantabria	4
CCAA	Castilla la Mancha	5
CCAA	Castilla León	6
CCAA	Cataluña	7
CCAA	Comunidad Valenciana	8
CCAA	Extremadura	9
CCAA	Galicia	10
CCAA	Islas Baleares	11
CCAA	Islas Canarias	12
CCAA	La Rioja	13
CCAA	Madrid	14
CCAA	Murcia	15
CCAA	Navarra	16
CCAA	País Vasco	17
CCAA	Ceuta	18
CCAA	Melilla	19
ZONA	Zona Sur: Extremadura + Andalucía + Islas Canarias	1
ZONA	Zona Cataluña + Aragón: Cataluña + Aragón	2
ZONA	Zona Levante: Com. Valenciana + Murcia + Baleares	3
ZONA	Zona Centro: Madrid + Castilla – La Mancha + Ávila + Segovia + Guadalajara	4
ZONA	Zona Norte: Galicia + Asturias + Cantabria + País Vasco + Navarra + La Rioja + León + Palencia + Burgos + Zamora + Valladolid + Soria + Salamanca	5
ZONA	Ceuta - Meilla	6
P2	Hombre	1
P2	Mujer	2
P4	Oncólogo médico	1
P4	Oncólogo médico residente	2
P4	Oncólogo radioterapeuta	3
P4	Cuidados paliativos	4
P4	Unidad del dolor	5
P4	Otra especialidad	6
P5	Hospital público	1
P5	Hospital privado	2
P7	GRUPO 1: Pequeño hospital comarcal, con menos de 150 camas de media, sin apenas dotación de alta tecnología, pocos médicos y escasa complejidad atendida	1
P7	GRUPO 2: Hospital general básico, tamaño medio menor de 200 camas, mínima dotación tecnológica, con algo de peso docente y algo mayor complejidad atendida	2
P7	GRUPO 3: Hospital de área, de tamaño medio en torno a 500 camas. Más de 50 médicos MIR y 269 médicos de promedio. Complejidad media (1,5 servicios complejos y 1,01 case mix)	3
P7	GRUPO 4: Grupo de gran hospital, pero más heterogéneos en dotación, tamaño y actividad. Gran intensidad docente (más de 160 MIR y elevada complejidad (4 servicios complejos de media y case mix mayor de 1,20)	4
P7	GRUPO 5: Hospital de gran peso estructural y mucha actividad. Oferta completa de servicios. Más de 680 médicos y en torno a 300 MIR. Incluye los grandes complejos	5

P12	Sí	1
P12	No	2
P22	Sí, siempre	1
P22	Casi siempre	2
P22	Algunas veces	3
P22	Nunca o casi nunca	4
P24	Sí, siempre	1
P24	Casi siempre	2
P24	Algunas veces	3
P24	Nunca o casi nunca	4

P20B_2_7	: RE +
P20B_2_8	: RE –
P20B_2_9	: RP +
P20B_2_10	: RP -
P20B_2_11	: Ki-67 Negative (=20%)
P20B_2_12	: Ki-67 Positivo (>20%)
P20B_2_13	: TILS = 30%
P20B_2_14	: TILS > 30%
P20B_2_15	: Paciente = 35 años
P20B_2_16	: Paciente = 35 años
P20B_2_17	: Paciente con comorbilidades
P20B_2_18	: Otra característica: Especificar:
P20B_2_COD	P20B_2 - Otra característica: Especificar:
P21_1	Estadio cN1: Neoadyuvancia: Quimioterapia multiagente + trastuzumab + pertuzumab
P21_2	Estadio cN1: Neoadyuvancia: Paclitaxel + trastuzumab + pertuzumab
P21B_1_1	: Tamaño del tumor =2 mm - =5 mm (T1a)
P21B_1_2	: Tamaño del tumor =6 mm- =10 mm (T1b)
P21B_1_3	: Tamaño del tumor =11 mm- =20 mm (T1c)
P21B_1_4	: Tamaño del tumor entre =20 mm
P21B_1_5	: Tamaño del tumor =21 mm- =30 mm
P21B_1_6	: Tamaño del tumor =31 mm- =50 mm
P21B_1_7	: Grado histológico I
P21B_1_8	: Grado histológico II
P21B_1_9	: Grado histológico III
P21B_1_10	: RE +
P21B_1_11	: RE –
P21B_1_12	: RP +
P21B_1_13	: RP -
P21B_1_14	: Ki-67 Negative (=20%)
P21B_1_15	: Ki-67 Positivo (>20%)
P21B_1_16	: TILS = 30%
P21B_1_17	: TILS > 30%
P21B_1_18	: Paciente = 35 años
P21B_1_19	: Paciente = 35 años
P21B_1_20	: Paciente con comorbilidades
P21B_1_21	: Otra característica: Especificar:
P21B_1_COD	P21B_1 - Otra característica: Especificar:

P21B_2_1	: Tamaño del tumor =2 mm - =5 mm (T1a)
P21B_2_2	: Tamaño del tumor =6 mm- =10 mm (T1b)
P21B_2_3	: Tamaño del tumor =11 mm- =20 mm (T1c)
P21B_2_4	: Tamaño del tumor entre =20 mm
P21B_2_5	: Tamaño del tumor =21 mm- =30 mm
P21B_2_6	: Tamaño del tumor =31 mm- =50 mm
P21B_2_7	: Grado histológico I
P21B_2_8	: Grado histológico II
P21B_2_9	: Grado histológico III
P21B_2_10	: RE +
P21B_2_11	: RE –
P21B_2_12	: RP +
P21B_2_13	: RP -
P21B_2_14	: Ki-67 Negative (=20%)
P21B_2_15	: Ki-67 Positivo (>20%)
P21B_2_16	: TILS = 30%
P21B_2_17	: TILS > 30%
P21B_2_18	: Paciente = 35 años
P21B_2_19	: Paciente = 35 años
P21B_2_20	: Paciente con comorbilidades
P21B_2_21	: Otra característica: Especificar:
P21B_2_COD	P21B_2 - Otra característica: Especificar:
P22	
P23_1	Régimen neoadyuvante en cT1a/b cN0: Paclitaxel x 12 + trastuzumab
P23_2	Régimen neoadyuvante en cT1a/b cN0: Paclitaxel x 12 + trastuzumab + pertuzumab
P23_3	Régimen neoadyuvante en cT1a/b cN0: Quimioterapia multiagente + trastuzumab
P23_4	Régimen neoadyuvante en cT1a/b cN0: Quimioterapia multiagente + trastuzumab + pertuzumab
P23_5_COD	Régimen neoadyuvante en cT1a/b cN0: Otra. Especificar: ...
P23_5	Régimen neoadyuvante en cT1a/b cN0: Otra. Especificar:
P24	
P25_1	Régimen neoadyuvante en cT1c cN0: Paclitaxel x 12 + trastuzumab
P25_2	Régimen neoadyuvante en cT1c cN0: Paclitaxel x 12 + trastuzumab + pertuzumab
P25_3	Régimen neoadyuvante en cT1c cN0: Quimioterapia multiagente + trastuzumab
P25_4	Régimen neoadyuvante en cT1c cN0: Quimioterapia multiagente + trastuzumab + pertuzumab
P25_5_COD	Régimen neoadyuvante en cT1c cN0: Otra. Especificar: ...
P25_5	Régimen neoadyuvante en cT1c cN0: Otra. Especificar:
P26_1	: Tamaño del tumor =2 mm - =5 mm (T1a)
P26_2	: Tamaño del tumor =6 mm- =10 mm (T1b)
P26_3	: Tamaño del tumor =11 mm- =20 mm (T1c)
P26_4	: Grado histológico I
P26_5	: Grado histológico II
P26_6	: Grado histológico III
P26_7	: RE +
P26_8	: RE –
P26_9	: RP +
P26_10	: RP –

P26_11	: Ki-67 Negative (=20%)	
P26_12	: Ki-67 Positivo (>20%)	
P26_13	: TILS = 30%	
P26_14	: TILS > 30%	
P26_15	: Paciente = 35 años	
P26_16	: Paciente = 35 años	
P26_17	: Paciente con comorbilidades	
P26_18	: Otra característica: Especificar:	
P26_COD	P26 - Otra característica: Especificar:	
P27		
P28_1	Trastuzumab 6 meses en adyuvancia para pacientes HER2+ subtipo pT1a pN0T1N0	
P28_2	Trastuzumab 6 meses en adyuvancia para pacientes HER2+ subtipo pT1b pN0T1N0	
P28_3	Trastuzumab 6 meses en adyuvancia para pacientes HER2+ subtipo pT1c pN0T1N0	
P28_4	Trastuzumab 6 meses en adyuvancia para pacientes HER2+ subtipo pT2 pN0T1N0	
P29		
P29	Sí	1
P29	No	2
P29_1	¿En qué pacientes?	
P29_2_1	¿Por qué?: Es una pauta que no está recogida en las principales guías clínicas/no cuenta con aprobación	
P29_2_2	¿Por qué?: No me siento seguro con una pauta a 6 meses	
P29_2_3	¿Por qué?: Los beneficios de trastuzumab 12 meses son superiores al riego de no pautarlo	
P29_2_4	¿Por qué?: Trastuzumab 12 meses es un tratamiento con efectos adversos manejables y/o que me preocupan poco	
P29_2_5	¿Por qué?: Otras. Especificar:	
P29_2_COD	P29_2 - Otras. Especificar:	
P30_1	Impacto del T-DM1 en adyuvancia para paciente HER2+ estadio clínico cT1 cN0 tratados con neoadyuvancia y enfermedad residual	
P31		
P31_COD	P31 - Otras. Especificar	
P32		
P32_COD	P32 - Otras. Especificar	
P33		
P33	Sí, siempre	1
P33	Casi siempre	2
P33	Algunas veces	3
P33	Nunca o casi nunca	4
CONTROL2		
P34_1	Predicción del valor pronóstico de las pacientes con cáncer de mama HER2+ en estadios precoces	
P34_2	Probabilidad de respuesta patológica completa tras tratamientos neoadyuvantes basados en terapia anti-HER2 de las pacientes con cáncer de mama HER2+ en estadios precoces	
P35_1	Puntuación del riesgo pronóstico	
P35_2	Puntuación de la probabilidad de pCR	
P35_3	Niveles de ARNm de ERBB2	
P36_1	En la actualidad, HER2DX puede ayudarme a tomar decisiones de tratamiento en pacientes seleccionadas	

P36_2	Es necesario que las directrices internacionales (ASCO, StGallen, ESMO, NCCN) recomienden el uso de HER2DX para que yo tome ciertas decisiones de tratamiento en determinadas pacientes seleccionadas
P36_3	Es necesaria la validación de HER2DX con un estudio prospectivo para que yo tome ciertas decisiones de tratamiento en determinadas pacientes seleccionadas
P36_4	La primera indicación de HER2DX debería ser para guiar el de-escalado de la duración de trastuzumab en determinadas pacientes seleccionadas
P36_5	La primera indicación de HER2DX debería ser para guiar el de-escalado de pertuzumab en determinadas pacientes seleccionadas
P36_6	La primera indicación de HER2DX debería ser para guiar el de-escalado en adyuvancia de T-DM1 en pacientes determinadas seleccionadas
P36_7	La primera indicación de HER2DX debería ser para guiar el de-escalado de quimioterapia multiagente en determinadas pacientes seleccionadas
P37_1	El dato de “risk score” ofrecido por HER2DX me ayudaría a tomar decisiones sobre si empezar con un tratamiento neoadyuvante o ir a cirugía primaria en aquellas pacientes cT1 cN0
P37_2	El dato de “pCR score” ofrecido por HER2DX me ayudaría a tomar decisiones sobre si empezar con un tratamiento neoadyuvante o ir a cirugía primaria en aquellas pacientes cT1 cN0
P37_3	El dato de “risk score” ofrecido por HER2DX me ayudaría a tomar decisiones sobre si empezar con un tratamiento neoadyuvante o ir a cirugía primaria en aquellas pacientes cT2 cN0
P37_4	El dato de “pCR score” ofrecido por HER2DX me ayudaría a tomar decisiones sobre si empezar con un tratamiento neoadyuvante o ir a cirugía primaria en aquellas pacientes cT2 cN0
P37_5	El dato ofrecido por HER2DX me ayudaría a tomar decisiones sobre el tratamiento de neoadyuvancia con solo paclitaxel + trastuzumab + pertuzumab (en vez de quimioterapia multiagente) en aquellas pacientes cT2 cN0
P37_6	El dato ofrecido por HER2DX me ayudaría a tomar decisiones sobre el tratamiento de neoadyuvancia con solo paclitaxel + trastuzumab + pertuzumab (en vez de quimioterapia multiagente) en aquellas pacientes cT2 cN1
P37_7	El dato ofrecido por HER2DX me ayudaría a tomar decisiones sobre el tratamiento de neoadyuvancia con solo paclitaxel + trastuzumab + pertuzumab (en vez de quimioterapia multiagente) en aquellas pacientes cT1 cN1
P37_8	El dato ofrecido por HER2DX me ayudaría a tomar decisiones sobre el tratamiento con trastuzumab 6 meses en adyuvancia para pacientes pT1a pN0
P37_9	El dato ofrecido por HER2DX me ayudaría a tomar decisiones sobre el tratamiento con trastuzumab 6 meses en adyuvancia para pacientes pT1b pN0
P37_10	El dato ofrecido por HER2DX me ayudaría a tomar decisiones sobre el tratamiento con trastuzumab 6 meses en adyuvancia para pacientes pT1c pN0
P37_11	El dato ofrecido por HER2DX me ayudaría a tomar decisiones sobre el tratamiento con TDM-1 en adyuvancia en determinados perfiles de pacientes con enfermedad residual tras tratamiento neoadyuvante
P37_12	El dato ofrecido por HER2X me ayudaría a no pautar pertuzumab adyuvante en pacientes de bajo riesgo por HER2DX que alcanzan una pCR tras tratamiento neoadyuvante.
P37_13	El dato ofrecido por HER2DX me ayudaría a no pautar ninguna quimioterapia en tumores pequeños de bajo riesgo por HER2DX en pacientes seleccionadas
