# Supplementary material for: Management of early-stage HER2-positive breast cancer and attitudes towards HER2DX test in Spain: insights from a nationwide survey
Source: Clin Transl Oncol. 2024 Apr 23;26(8):2060–9. doi: 10.1007/s12094-024-03409-4 (PMC11249709; doi:10.1007/s12094-024-03409-4)
Supplement: Supplementary file 2 — Supplementary file1 (PDF 440 KB) [file 12094_2024_3409_MOESM2_ESM.pdf]

Supplementary Data

Definitions for groups according to size of the hospital

GROUP 1: A small regional hospital, averaging less than 150 beds, equipped with minimal high-tech facilities, and limited medical staff, resulting in relatively straightforward care.

GROUP 2: A basic general hospital, with fewer than 200 beds, offering limited technological resources, some involvement in medical education, and a slightly higher complexity in patient care.

GROUP 3: An area hospital of medium size, housing around 500 beds. It boasts a significant number of resident doctors (more than 50) and an average of 269 doctors. The complexity of medical services is moderate, with 1.5 complex services and a case mix of 1.01.

GROUP 4: A group of large hospitals, showcasing diversity in equipment, size, and activity. These hospitals have a strong emphasis on medical education with over 160 resident doctors and provide high-complexity medical services, averaging 4 complex services with a case mix greater than 1.20.

GROUP 5: A hospital with considerable structural significance and high activity. It offers a comprehensive range of services and has a substantial medical staff, with over 680 doctors and approximately 300 resident doctors. This category includes the large healthcare complexes.

Supplementary figures

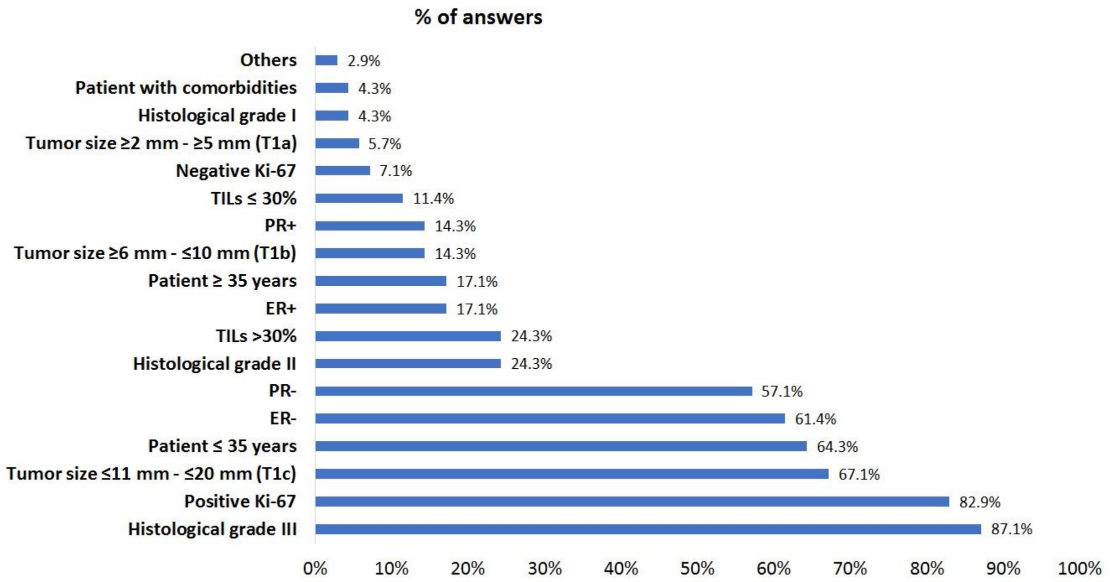

Figure S1. Factors that would make consider adjuvant multiagent chemotherapy in patients with pathological stage 1 (pT1 N0) disease. RE, estrogen receptor; PR;

progesterone receptor; TILs, tumor infiltrating lymphocytes. Ki-67 positive was defined as >20%; Ki-67 negative was defined as ≤20%.

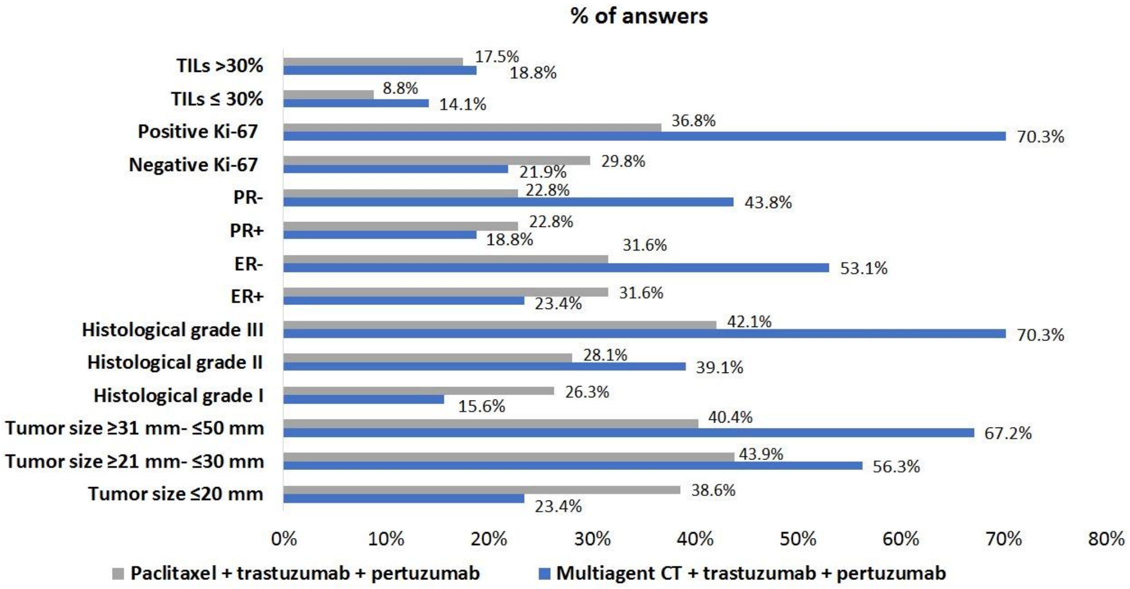

Figure S2. Factors that would make consider neoadjuvant single agent versus multiagent chemotherapy in patients with clinical stage T2 N0 disease. TILs, tumor infiltrating lymphocytes; RE, estrogen receptor; PR; progesterone receptor. Ki-67 positive was defined as >20%; Ki-67 negative was defined as ≤20%.

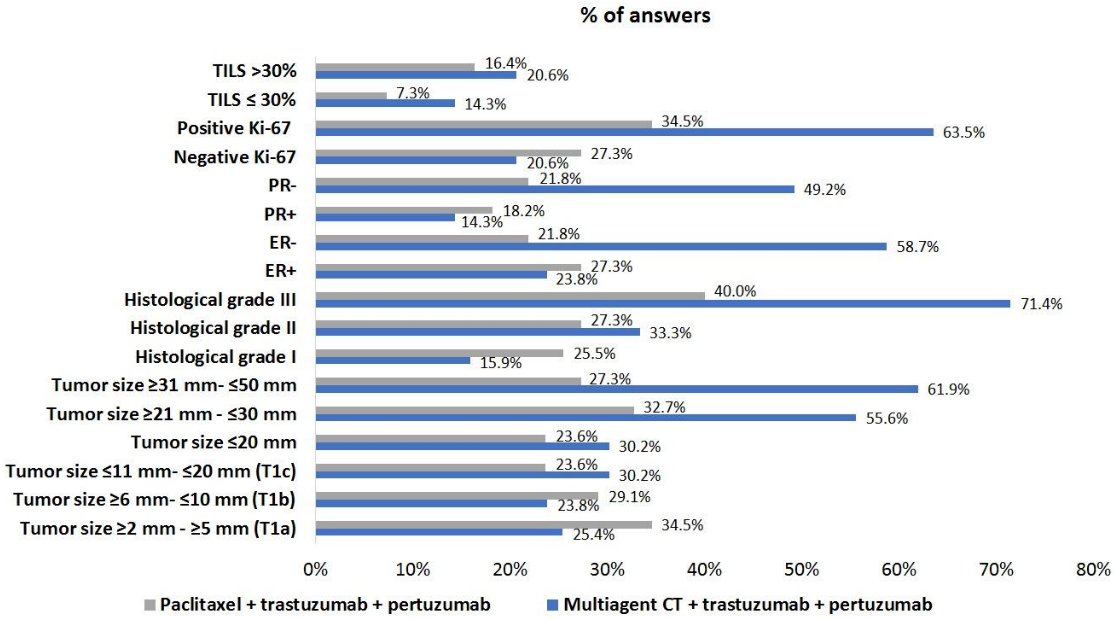

Figure S3. **Factors that would make consider neoadjuvant single agent versus multiagent chemotherapy in patients with clinical N1 disease**. TILs, tumor infiltrating

lymphocytes; RE, estrogen receptor; PR; progesterone receptor. Ki-67 positive was defined as >20%; Ki-67 negative was defined as ≤20%.
